# Detection of Synaptic Vesicle Glycoprotein 2A in Serum Using a Polypyrrole-Functionalized Graphene Oxide Electrochemical Immunosensor

**DOI:** 10.3390/nano16070397

**Published:** 2026-03-25

**Authors:** Yonghong Zhao, Le Li, Jiale Tao, Manying Yang, Chen Li, Xiaoqian Zhang, Yang Zhang, Shiguo Sun, Na Zhao

**Affiliations:** 1 Pharmacy College of Shihezi University/Key Laborataty of Xinjiang Phytomedicine Resource and Utilization, Ministry of Education/Collaborative Innovation Center for Efficient Safflower Production and Resource Utilization of XPCC/Institute for Safflower Industry Research, Shihezi University, Shihezi 832002, China; 20232015022@stu.shzu.edu.cn (Y.Z.); 20242015005@stu.shzu.edu.cn (J.T.); yangmanying@stu.shzu.edu.cn (M.Y.); lichen1@stu.shzu.edu.cn (C.L.); 20232015021@stu.shzu.edu.cn (X.Z.); 20252015015@stu.shzu.edu.cn (Y.Z.); 2 College of Chemistry & Pharmacy, Northwest A&F University, Xianyang 712100, China; teacher.lee@foxmail.com

**Keywords:** Alzheimer’s disease, immunosensor, conductive polymer, synaptic vesicle glycoprotein 2A

## Abstract

Early intervention is pivotal for mitigating the progression of Alzheimer’s disease (AD). This study presents an electrochemical immunosensor targeting synaptic vesicle glycoprotein 2A (SV2A) to facilitate early AD diagnosis. A sensing interface was engineered using a nanocomposite of graphene oxide (GO) and 3-carboxyl polypyrrole (3-COOH-PPy). Leveraging the synergistic effects between the large specific surface area of GO and the superior conductivity of 3-COOH-PPy, the composite established an efficient electron transport network. This architecture provided abundant active sites for capture antibody immobilization while significantly enhancing interfacial electron transfer kinetics. Coupling this interface with an enzyme-mediated signal amplification strategy based on the horseradish peroxidase (HRP)-catalyzed TMB/H_2_O_2_ system, the immunosensor achieved high sensitivity. It exhibited a wide linear range of 2 ng/mL to 16 μg/mL with a low limit of detection (LOD) of 0.15 ng/mL. Furthermore, successful detection in C57 mouse serum samples validated the method’s reliability and potential for clinical application. In conclusion, this immunosensor offers a sensitive and robust platform for the early diagnosis of AD.

## 1. Introduction

Alzheimer’s disease (AD) is the most prevalent age-related progressive neurodegenerative disorder. Accumulating evidence indicates that patients with AD begin to experience pathological changes and neuropathological alterations 10–20 years before the initial appearance of symptoms [1]. Currently, there are no curative drugs available for Alzheimer’s disease in clinical practice. Therefore, early intervention is widely regarded as a pivotal strategy for delaying disease progression and enhancing the quality of life for patients. Achieving this goal is contingent upon highly sensitive and specific biomarkers for early diagnosis [2]. However, although biomarkers such as Aβ42/Aβ40, phosphorylated tau (p-tau), neurofilament light (NfL), and glial fibrillary acidic protein (GFAP) have demonstrated certain efficacy in the early diagnosis of AD [3,4,5,6,7,8], effective blood-based biomarkers for early warning and diagnosis have not yet been established. Synaptic loss and synaptic dysfunction are primary mechanisms underlying the pathological basis of mild cognitive impairment (MCI) in early AD and the structural substrate of AD dementia [9]. Synaptic vesicle glycoprotein 2A (SV2A) is an essential vesicle membrane protein ubiquitously expressed in synapses; it participates in synaptic vesicle trafficking, exocytosis, and neurotransmitter release, and regulates gene and protein expression. Consequently, SV2A is considered the first in vivo marker of synaptic density [10,11]. Wang et al. [10] reported that SV2A levels in both cerebrospinal fluid and serum are significantly positively correlated with cognitive performance in AD patients, and that these levels decrease gradually as AD progresses. Furthermore, serum SV2A demonstrated excellent diagnostic performance for amnestic mild cognitive impairment (aMCI), with a sensitivity of 97.8%, which is significantly higher than that of NfL, GFAP, and p-tau217. The aforementioned studies suggest that SV2A holds potential as an early diagnostic biomarker and therapeutic target for AD.

Numerous studies have utilized synaptic positron emission tomography (Synaptic PET) tracers to assess SV2A levels in the hippocampus of participants. For instance, PET studies using the SV2A radiotracer 11C-UCB-J have demonstrated that SV2A binding in the hippocampus is significantly reduced in AD patients compared to healthy controls [12]. However, PET imaging is limited by its high cost, radiation risks, and the challenges associated with large-scale repetitive screening. Immunosensors, utilizing the specific binding between antibodies and antigens, can be employed to detect biomarkers for various diseases [13]. They offer a robust platform in the quantitative determination of biomarkers due to their operational simplicity and high sensitivity. Nevertheless, given the low abundance of in vivo biomarkers, which are often present in trace amounts, researchers typically focus on increasing the specific surface area of materials and improving electrical conductivity to enhance the sensitivity of electrochemical sensors [14,15]. Graphene oxide (GO) is a typical carbon nanomaterial characterized by a large specific surface area, abundant active sites, and excellent stability and biocompatibility; it has been applied in the construction of highly sensitive biosensors [16,17]. Among electrochemical-conductive polymers, polypyrrole (PPy) is an excellent electrode material characterized by ease of synthesis, good conductivity, and environmental friendliness [18]. However, its high rigidity and poor mechanical ductility [19] necessitate combination with other materials to form composite biosensors. These composite sensors synergize the advantages of conductive polymers with other materials, offering higher sensitivity and stability, making them suitable for various complex bio-detection tasks [20,21]. He et al. [22] fabricated a hierarchical composite of reduced graphene oxide and platinum-nickel-decorated polyaniline nanospheres (rGO/PANI@PtNi), which demonstrated highly sensitive detection for quantifying hydrogen. Zhao et al. [23] constructed a sensor for detecting amyloid-beta oligomers (AβO) by embedding gold nanoparticles (AuNPs) into a poly(pyrrole-3-carboxylic acid) conductive polymer matrix, significantly improving sensor sensitivity by leveraging the large specific surface area and high conductivity of the conductive polymer. Shi et al. [24] integrated gold nanoparticles (AuNPs) and polypyrrole (PPy) hydrogel into a foldable paper-based system to achieve high-sensitivity detection of COVID-19. Currently, there are few reports on electrochemical sensing methods for detecting SV2A in the blood of AD patients.

In this paper, an electrochemical immunosensor based on 3-COOH-PPy/GO nanocomposites modified glassy carbon electrode (GCE) was designed for the detection of SV2A, a biomarker associated with Alzheimer’s disease (AD). By electrodepositing 3-COOH-PPy onto the GO surface, the sensor leverages the synergistic effect of the large specific surface area of GO and the excellent conductivity of PPy to significantly enhance the electron transport performance at the electrode surface. Simultaneously, the abundant carboxyl groups of 3-COOH-PPy served as active sites to achieve efficient immobilization of capture antibodies. Regarding the detection strategy, an enzyme-mediated signal amplification mechanism employing HRP to catalyze the TMB/H_2_O_2_ system was adopted, enabling highly sensitive detection of SV2A as a model target. Furthermore, spike-and-recovery experiments in healthy C57 mouse serum further validated the practical applicability of the sensor. The results indicate that the 3-COOH-PPy/GO/GCE sensor holds significant potential for the in vitro detection of AD biomarkers.

## 2. Experimental

### 2.1. Materials and Methods

C-MAG HS4 constant temperature magnetic stirrer (IKA, Staufen, Germany); centrifuge (Xiangyi Centrifuge Instrument Co., Ltd., Changsha, China); scanning electron microscope (Carl Zeiss, Oberkochen, Germany, Zeiss Sigma 300); CHI 1040C electrochemical workstation (Chenhua Instrument Co., Ltd., Shanghai, China); ES225SM-DR electronic analytical balance (Prieses (Shanghai) Co., Ltd., China); WP-USP-40 laboratory ultrapure water machine (Water Technology Equipment Co., Ltd., Chengdu, China); electric thermostatic blast drying oven (Jinghong Scientific Instruments Co., Ltd, Shanghai, China).

Bovine serum albumin (BSA) was supplied by Shanghai Energy Chemical (Shanghai, China). SV2A antibody and SV2A protein were both purchased from Abcam. 3-(3-Dimethylaminopropyl)-1-ethylcarbodiimide hydrochloride (EDC), N-hydroxysuccinimide (NHS), 3,3′,5,5′-tetramethylbenzidine (TMB, ≥98%), hydrogen peroxide (30% H_2_O_2_), 3-carboxypyrrole (Cat. 99%), potassium ferrocyanide (K_4_Fe(CN)_6_·3H_2_O, ≥98.5%), potassium chloride (KCl, Cat. 99%), and phosphate-buffered saline (PBS) were purchased from Shanghai Aladdin Biochemical Technology Co., Ltd. (Shanghai, China). Potassium ferricyanide (K_3_Fe(CN)_6_) was purchased from Nacalai Tesque Co., Ltd. (Tokyo, Japan). DuPont Nafion solution (resin solid content: 20%, water content: 34%, volatile organic compound content: 46%, ion exchange capacity: 1.03–1.12) was purchased from Shanghai Jingchong Electronic Technology Development Co., Ltd. (Shanghai, China). The water used for all experiments was ultrapure water obtained from the aforementioned ultrapure water system. All chemicals were of analytical grade and were used without further purification.

### 2.2. Preparation of Graphene Oxide

The preparation of graphene oxide (GO) primarily followed an improved Hummers method [25]: 18 mL of sulfuric acid and 2 mL of phosphoric acid (at a volume ratio of 9:1) were mixed and stirred for 15 min. Then, 0.1485 g of graphite flakes were added, followed by 1 mL of phosphoric acid to rinse the walls of the round-bottom flask, and the mixture was stirred for 3 min. Subsequently, 0.8722 g of potassium permanganate was slowly added, and the reaction was stirred for 6 h (the solution appeared dark green). Finally, 0.225 mL of hydrogen peroxide was added to remove unreacted potassium permanganate. The reaction solution was washed and dried to obtain the product.

### 2.3. Preparation of 3-COOH-PPy/GO/GCE Sensor

First, a bare GCE with a diameter of 5 mm was polished several times using 0.30 mm and 0.05 mm alumina powders until a mirror-like finish was achieved. The electrode was then placed in a mixed solution of ultrapure water and ethanol for 3 min, and ultrasound was applied to remove physically adsorbed substances. Second, 1 mg of graphene oxide powder was added to 1 mL of deionized water, followed by the addition of 4 μL of 0.5% Nafion solution, and the mixture was ultrasonicated to obtain a homogeneous suspension. Subsequently, 10 μL of the aforementioned suspension was dropped onto the surface of the freshly polished GCE and dried under a daylight lamp.

**Scheme 1:**
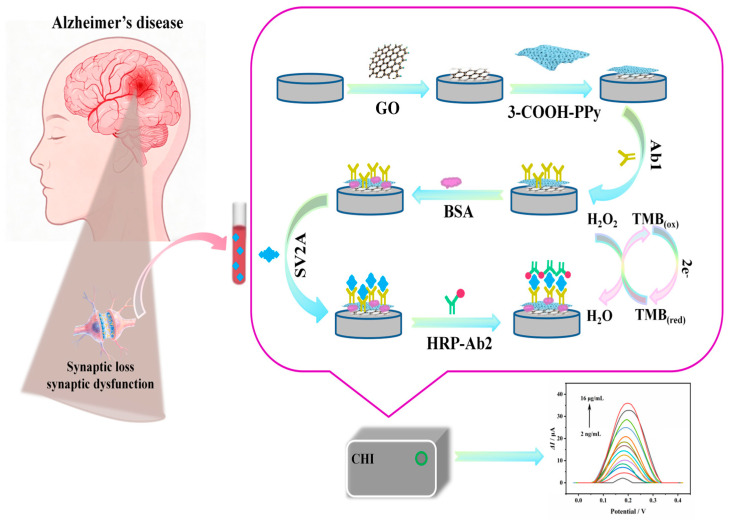
Diagram of the detection of SV2A based on 3-COOH-PPy/GO composite material.

The GO/GCE was immersed in a 0.04 mol/L 3-carboxylic acid pyrrole monomer aqueous solution, and then electropolymerization was carried out in a 0.5 mol/L sulfuric acid solution using the CV method. The scanning potential ranged from −1.3 to 0.85 V, with a scanning rate of 50 mV/s for 3 consecutive cycles. The prepared electrode was dried with nitrogen and designated as 3-COOH-PPy/GO/GCE. The nanocomposite-modified electrode was immersed in a PBS solution (0.01 M, pH 7.40) containing 30 mmol/L EDC and 30 mmol/L NHS for 70 min to activate the carboxyl groups on the composite surface. It was stored at 4 °C for further use. The whole fabrication processes of the electrochemical immunosensor were described in Scheme 1.

### 2.4. Preparation of the Electrochemical Immunosensor

Subsequently, 10 μL of SV2A antibody (Ab1) at a concentration of 1 μg/mL was drop-cast onto the surface of the activated 3-COOH-PPy/GO/GCE and incubated overnight at 4 °C. Next, 10 μL of 0.1 wt% bovine serum albumin (BSA) solution was applied to the electrode surface to block non-specific sites for 1 h. Following this, the BSA-blocked electrode was immersed in an SV2A protein solution at room temperature for 40 min. Finally, 50 μL of HRP-Ab2 solution (1 μg/mL) was dropped onto the electrode and incubated at room temperature for 1 h. Each step was followed by washing with PBS solution (0.01 M, pH 7.4) to remove unbound antibodies and proteins. The current response to a 30 μg/mL TMB/H_2_O_2_ substrate solution was measured using differential pulse voltammetry (DPV). The DPV parameters were set as follows: potential range, −0.4 to 1.2 V; pulse amplitude, −48 to −50 mV; step potential, 5 mV; pulse width, 0.1 s; and scan rate, 50 mV/s.

### 2.5. Analysis in Serum Samples

Venous blood was obtained from healthy C57 mice. The collected blood samples were allowed to stand undisturbed in a refrigerator for 2 h, and then processed using a low-temperature centrifuge at 3000 r/min for 15 min. The supernatant was isolated, sealed, and stored at −80 °C until use. Prior to analysis, the serum samples were diluted 1:50 (*v*/*v*) with PBS to mitigate matrix effects. Throughout the course of this experiment, all ethical regulations were strictly observed in accordance with the Declaration of Helsinki.

## 3. Results and Discussion

### 3.1. Characterization of the 3-COOH-PPy/GO Nanocomposites

**Figure 1:**
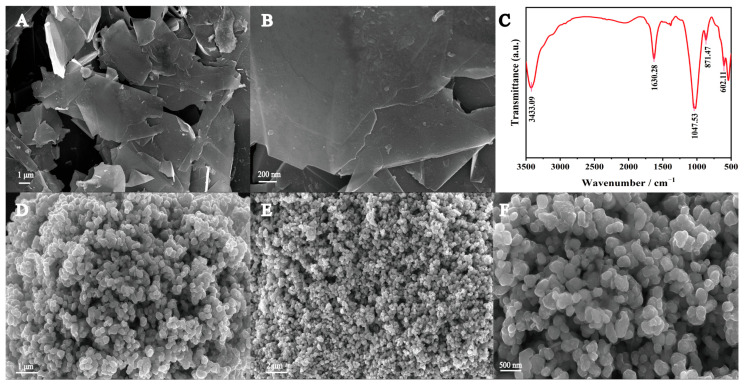
(**A**,**B**): SEM images of GO; (**C**): the FTIR image of GO; (**D**): SEM image of 3-COOH-PPy; (**E**): SEM image of 3-COOH-PPy/GO; (**F**): magnified SEM image of 3-COOH-PPy/GO.

SEM and FTIR were employed to verify the successful preparation of GO. The results are shown in Figure 1A,B below; the prepared GO exhibits a sheet-like structure, which is consistent with reports [26]. The FTIR results (Figure 1C) show that the prepared GO has a strong and broad absorption peak at 3433.09 cm^−1^, attributed to the stretching vibration peak of -OH; a peak at 1630.28 cm^−1^ corresponds to the stretching vibration of C=O; and a peak at 1047.53 cm^−1^ corresponds to the vibration absorption of C-O-C. These findings indicate that GO contains -COOH groups. The free -COOH groups can form amide bonds with antibodies, thereby facilitating antibody immobilization.

**Figure 2:**
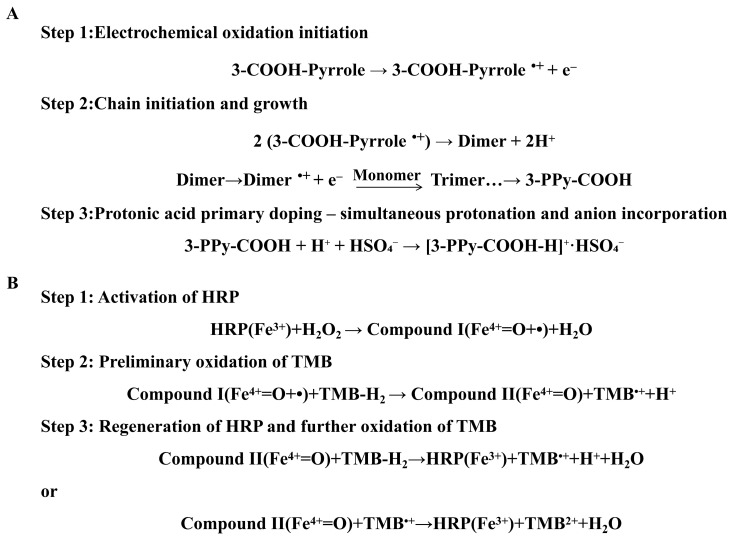
(**A**) Mechanism diagram of electropolymerization of 3-COOH-PPy in the presence of 0.5 mol/L H_2_SO_4_. (**B**) Mechanism diagram of the HRP catalyzing TMB/H_2_O_2_ system.

Figure 1D presents the SEM image of the 3-COOH-PPy nanoparticles, revealing large spherical nanoparticles with a uniform particle size distribution. A typical cauliflower-like structure can be observed under SEM. Electropolymerization was used to polymerize 3-COOH-PPy on the surface of GO. For CV polymerization, the generally accepted mechanism is that during the polymerization of polypyrrole, the monomer first undergoes oxidation and then fuses with another radical cation. This leads to the loss of two protons, forming a dimer radical cation. These dimers then undergo further oxidation and combine with radical cations, promoting the growth of the polymer chain. This process continues until the polymer chain reaches the expected length [26] (Figure 2A). It is noteworthy that the presence of protons (derived from H_2_SO_4_) not only facilitates the radical-cation-initiated chain polymerization of 3-COOH-PPy but also participates in the primary doping process. During this process, the protonation of the polymer backbone occurs simultaneously with the incorporation of bisulfate anions. This synergistic step introduces charge carriers while compensating for the positive charges via anions, thereby endowing the polymer with high electrical conductivity [27]. Figure 2A illustrates this synchronous doping mechanism involving hydrogen ions (protons) and bisulfate ions (anions), explicitly elucidating their inseparable synergistic effects in constructing the conductive polymer chains and maintaining charge neutrality. Figure 1E,F displays the prepared conductive polymer growing uniformly on the GO surface, which significantly increases the surface area of GO, provides binding sites for antibodies but ‘redox active sites for electron transfer’ with solution-phase mediators. This structure is frequently observed in PPy materials obtained via electropolymerization and appears to cover the surface uniformly. These results indicate that the conditions of the electropolymerization process are favorable for the formation of 3-COOH-PPy. Although direct spectroscopic evidence was not obtained due to experimental constraints, the significant changes in electrochemical impedance and the morphological differences observed in SEM images provided indirect support for the formation of the composite structure.

### 3.2. Electrochemical Characterization of 3-COOH-PPy/GO/GCE

**Figure 3:**
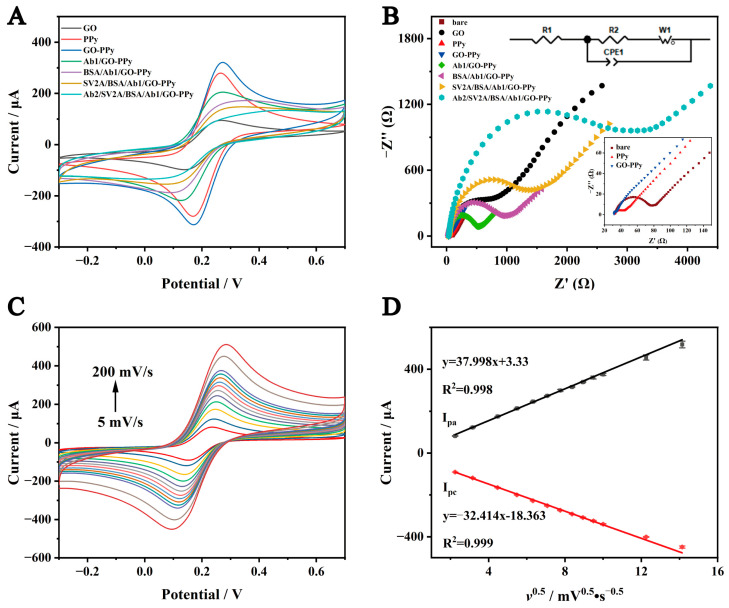
(**A**) CV diagram of different electrodes in a 5 mmol/L [Fe(CN)_6_]^3−/4−^ solution. (**B**) EIS diagram of different electrodes in 5 mmol/L [Fe(CN)_6_]^3−/4−^ solution. (**C**) CV curves of 3-COOH-PPy/GO/GCE under different scan rates (5–200 mV/s) in 5 mmol/L [Fe(CN)_6_]^3−/4−^ solution. (**D**) Linear relationship between peak intensity in (**C**) and the square root of scan rate (*n* = 3).

Electrochemical characterization was primarily conducted using cyclic voltammetry (CV) and electrochemical impedance spectroscopy (EIS) to verify the interfacial properties of the electrode following each modification step. In this experiment, 5 mmol/L [Fe(CN)_6_]^3−/4−^ was used as the electrolyte within a conventional three-electrode system, utilizing a glassy carbon electrode (GCE) as the working electrode, a platinum wire electrode as the counter electrode, and a saturated Ag/AgCl electrode as the reference electrode. Figure 3A displays the CV curves of the electrodes during different modification stages. As observed in the figure, the introduction of 3-COOH-PPy resulted in a significant increase in the redox peaks of [Fe(CN)_6_]^3−/4−^. This is attributed to the large specific surface area of the nanospherical structure formed by the 3-COOH-PPy conductive polymer, which provides binding sites for antibodies but ‘redox active sites for electron transfer’ with solution-phase mediators. With the immobilization of anti-SV2A and the subsequent capture of the SV2A protein, the redox peaks gradually decreased due to the non-conductive nature of the antibody-protein immunocomplex. These changes correspond one-to-one with the variations in charge-transfer resistance observed in the EIS spectra in Figure 3B, specific values are provided in Table 1, indicating the successful stepwise modification of the sensor. This indicates the successful stepwise modification of the sensor. The inset in Figure 2B illustrates Randles equivalent circuit model, which comprises four components: Rs, Cdl, Rct, and Zw, representing the electrolyte solution, capacitance, electron transfer resistance, and Warburg impedance, respectively.

To conduct a more comprehensive analysis of the electrochemical behavior of the proposed immunosensor, the sensor was incubated in 5 mmol/L [Fe(CN)_6_]^3−/4−^ for CV analysis. The scan rate was varied within the range of 5–200 mV/s to obtain useful insights into its reaction kinetics (Figure 3C,D). As the scan rate increased, the oxidation and reduction peak potentials shifted towards positive and negative directions, respectively, and the peak redox currents increased. Based on the quantitative relationship between peak currents and scan rates, the calibration curve equations for the redox peaks were determined to be: ipc (μA) = −30.627x + 30.217 (R^2^ = 0.996) and ipa (μA) = 35.884x + 13.498 (R^2^ = 0.998). Furthermore, the symmetric V-shape of the two curves in Figure 3D indicates that the 3-COOH-PPy/GO/GCE sensor possesses superior reversibility, facilitating faster and easier electron transfer at the sensing interface. These results suggest that the detection of antigen–antibody complexation is a diffusion-controlled redox process involving the inner-sphere redox mediator and the electrode surface [28]. It is well established that the voltametric current in CV curves is positively correlated with the effective active surface area (A_eff_) of the electrode [29]. According to the Randles–Sevcik equation [30], the electrochemically active surface area of the modified electrode can be calculated.

$$ip=(2.69\times 10^{5})n^{3/2}D^{1/2}v^{1/2}AC$$

In the equation, *ip*, *n*, *D*, *v*, *A*, and *C* represent the peak current (A), the number of transferred electrons, the diffusion coefficient of [Fe(CN)_6_]^3−/4−^ (7.6 × 10^−6^ cm^2^/s), the scan rate (V/s), the electrochemically active surface area of the modified electrode (cm^2^), and the concentration of [Fe(CN)_6_]^3−/4−^ (mol/cm^3^), respectively. The calculated A_eff_ of the composite material is 0.3872 cm^2^, which is significantly larger than that of individual materials (Table 1). This indicates that the high density of effective active sites endows the composite electrode material with superior electrocatalytic capability.

### 3.3. Optimization of Experimental Conditions

To determine the optimal experimental conditions for the SV2A immunosensor, the effects of the polymerization rate of 3-COOH-PPy (10, 25, 50, and 75 mV/s), the number of CV cycles for 3-COOH-PPy polymerization (1, 2, 3, 4), the concentration of the 3-carboxypyrrole monomer (0.01, 0.02, 0.04, 0.08 mol/L), the volume of cast nanocomposite (5, 8, 10, 12, and 15 μL), the BSA concentration (0.1%, 0.5%, 1.0%, and 2.0%), and the incubation time for the primary antibody with the SV2A protein (20, 40, 50, and 60 min) on the experimental results were investigated. The current changes were recorded using DPV to establish the optimal conditions.

**Figure 4:**
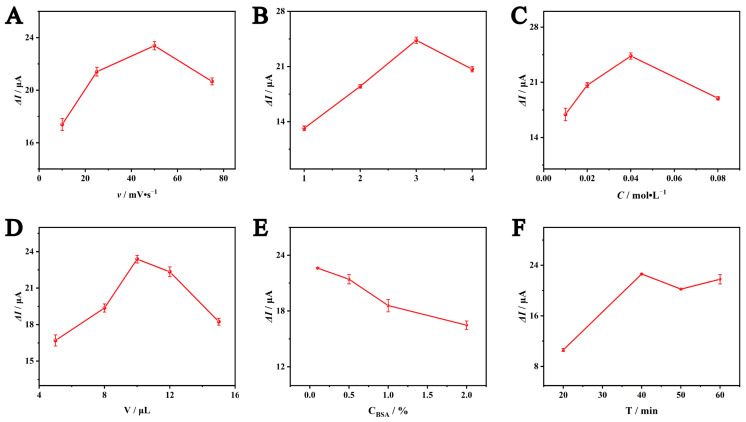
Optimization of electrode preparation conditions and performance evaluation. (**A**). Analysis of the polymerization rate during the electropolymerization of 3-COOH-PPy. (**B**). The number of cyclic voltammetry (CV) scans required for the electropolymerization of 3-COOH-PPy. (**C**). The concentration of 3-COOH-pyrrole monomer; The influence on the differential pulse voltammetry (DPV) response of 3-COOH-PPy/GO electrode. (**D**). The effect of the modification amount of 3-COOH-PPy/GO on the DPV response characteristics of the electrode. (**E**). The effect of BSA sealing concentration on the binding efficiency of Ab1 and SV2A. (**F**). The incubation time of Ab1 and SV2A; The influence on the current response of SV2A at 1 μg/mL in PBS (0.01 M, pH 7.4). All experiments were repeated 3 times.

Figure 4A illustrates the effect of different scan rates on the electropolymerization of 3-COOH-PPy. DPV responses suggest that scan rates between 10 and 50 mV/s facilitate the growth of a polymer layer within the GO porous network. Under these conditions, the electroactivity of the pore surface and polymer layer increases as they fully penetrate the interior of the porous electrode. However, further increasing the 3-COOH-PPy loading (at a scan rate of 75 mV/s) produces a thicker polymer layer, which may hinder penetration into the inner portions of the porous material, leading to a decrease in electroactivity. The CV cycles significantly influenced the formation of 3-COOH-PPy (Figure 4B). Within the range of 1 to 3 cycles, the response signal of the 3-COOH-PPy/GO composite towards TMB gradually increased with the increasing number of scanning cycles; this is attributed to the prolonged polymerization time, which increased the loading of the conductive polymer on the GO surface. However, when the CV cycles reached 4, the current response exhibited a downward trend, likely because excessive 3-COOH-PPy loading increased the created a steric barrier that impeded electron transport, thereby impeding electron transport. The influence of monomer concentration followed a similar trend (Figure 4C), with the formation of 3-COOH-PPy reaching an optimum at 0.04 mol/L. As shown in Figure 4D, the peak current of TMB increases with the increasing volume of the cast nanocomposite, confirming that 3-COOH-PPy/GO can effectively enhance sensitivity. However, the current significantly decreases when the modified volume exceeds 10 μL; this is attributed to the agglomeration of the nanocomposite on the GCE surface at higher volumes, which impedes electron transfer.

The concentration of BSA has a significant impact on the performance of the immunosensor, and its reasonable optimization is a critical step for improving sensor sensitivity, specificity, and stability. Figure 4E indicates that an excessively high BSA concentration may hinder the effective binding of the target antigen and antibody, thereby reducing detection sensitivity. As can be seen from Figure 4F, the subsequent current response stabilizes after incubating SV2A with the antibody for 40 min, indicating that the binding between SV2A and the antibody reaches saturation at 40 min. Therefore, the optimal capture time for SV2A is determined to be 40 min.

### 3.4. The Detection Performance of the Immunosensor for SV2A

**Figure 5:**
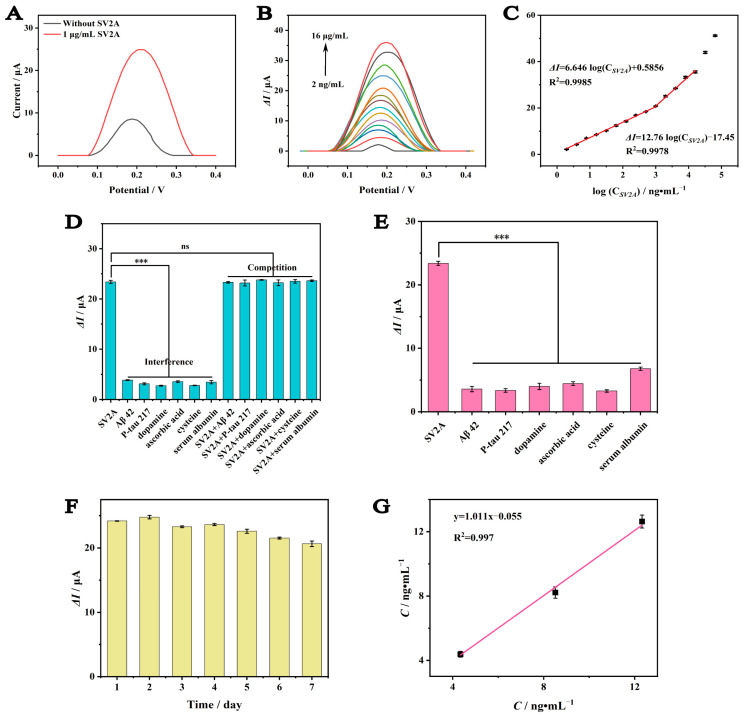
(**A**) The signal response of the sensor on the absence (black curve) and presence of 1 μg/mL SV2A (red curve) in 0.01 mol/L PBS. (**B**) DPV response of the electrochemical immunosensor after incubation with different concentrations of SV2A in PBS (pH 7.4). (**C**) The dependence of the DPV current signal on the logarithm of SV2A concentration within the range of 2 ng/mL to 16 μg/mL. (**D**) Study on the interferents factors of 1 μg/mL SV2A and some biomolecules. (**E**) Study of the interfering factors of 1 μg/mL SV2A and some biological molecules was conducted in a serum sample diluted 50 times. (**F**) The 4 °C storage stability of the prepared immunosensor. (**G**) Linear correlation graph of ELISA kit and immunosensor for the detection concentration of the same sample. All experiments were repeated 3 times. *** *p* < 0.001; ns, no significance.

Under optimal conditions, the electrochemical performance of the prepared 3-COOH-PPy/GO/GCE for SV2A analysis was further investigated. Theoretically, increasing concentrations of SV2A bring more HRP, which catalyzes more TMB in the presence of H_2_O_2_, resulting in a higher DPV current (Figure 2B). As shown in Figure 5A, compared with the control sensor without an amplification strategy, the signal intensity of the sensor based on the HRP-TMB/H_2_O_2_ amplification system was enhanced by approximately 3-fold, demonstrating the effectiveness of this enzyme-mediated amplification strategy. Figure 5B,C displays a piecewise linear relationship between the DPV current intensity and the logarithm of the SV2A concentration. In the range of 2–250 ng/mL, the linear equation is △*I* = 6.646 logC + 0.5856 (R^2^ = 0.9985); in the range of 0.25–16 μg/mL, the linear equation is △*I* = 12.76 logC − 17.45 (R^2^ = 0.9978), where △*I* represents the difference in DPV peak intensity between the target and the blank. This piecewise linear relationship significantly broadens the dynamic range, covering a wider spectrum of clinical sample concentrations and minimizing the need for sample dilution. Consequently, the sensor is suitable for both screening (low concentrations) and monitoring (high concentrations). It enables the POCT device to maintain high-precision quantification across a wider concentration range, thereby substantially enhancing its applicability in complex clinical scenarios. The limit of detection was determined to be 0.15 ng/mL by dividing three times the standard deviation of the blank sample by the slope of the calibration curve. This is lower than or comparable to the limits of detection calculated for other reported methods (Table 2) [31,32,33,34]. According to research data [10], the mean concentration of the healthy control group (~5.3 ng/mL) and the optimal cutoff value for diagnosing aMCI (5.05 ng/mL) both fall within the linear range, indicating that the sensor possesses high precision in distinguishing between healthy individuals and patients with early mild cognitive impairment, making it suitable for early screening of AD. Although the diagnostic cutoff value for AD patients (~1.67 ng/mL) is slightly below the lower limit of the linear range, the sensor still exhibits significant signal response to low-concentration samples due to its ultra-low limit of detection (0.15 ng/mL).

### 3.5. Selectivity, Stability, and Reproducibility

The reproducibility of the SV2A immunosensor was evaluated by assessing the DPV current response variation across six independently fabricated electrodes. Under identical experimental conditions, six GCEs were subjected to the same treatment to obtain electrodes modified with the 3-COOH-PPy/GO nanocomposite. The SV2A immunosensors were then prepared to detect a 1 μg/mL SV2A standard solution. The results showed that the DPV current values of the six independent electrodes were similar, with a relative standard deviation (RSD) of 3.58%, indicating that the SV2A immunosensor possesses good reproducibility.

The prepared SV2A immunosensors were stored at 4 °C and measured every day. As shown in Figure 5F, with increasing storage time, the current values measured by the sensor slowly decreased due to the declining activity of the 3-COOH-PPy/GO nanocomposite and the biomolecules. The current signal obtained via DPV for the modified electrodes remained at 85.39% of the initial value after 7 days of storage, and the RSD of the current values over 7 consecutive days was 6.38%. These results demonstrate that the prepared sensor exhibits acceptable stability (Figure 5F).

Prior to applying the sensing system to biological samples, the influence of various endogenous substances coexisting in blood on the detection performance of the immunosensor was evaluated. The selectivity of the electrochemical analysis system for SV2A was investigated using interference and competition experiments. Specifically, the effects of Aβ 42, P-tau 217, dopamine, ascorbic acid, cysteine, and serum albumin on SV2A detection were examined. The DPV signals of these interferents were measured separately, and their current signals when measured individually were compared with those when mixed with SV2A. As shown in Figure 5D, the current responses of the interfering substances were extremely weak and exhibited a significant difference compared to the current response of SV2A (*p* ≈ 3.3 × 10^−18^). Using the same method, the influence of the aforementioned interfering substances on SV2A detection was re-examined in 50-fold diluted serum from healthy C57 mice. The results, as shown in Figure 5E, indicate that even when the concentrations of interfering substances far exceeded that of SV2A (by more than 20 times), their DPV response signals remained less than 1/4 of the SV2A signal, showing a significant difference (*p* < 0.001). This is attributed to the high specificity of the anti-SV2A on the sensor towards SV2A. Furthermore, Figure 5D demonstrates that the electrochemical signals generated when the aforementioned interferents were mixed with SV2A were nearly identical to those obtained during individual detection. The *p* values, from left to right, were 0.822, 0.618, 0.102, 0.316, 0.135, and 0.250, all of which were >0.05, indicating no significant difference. This suggests that the presence of interfering substances does not affect the determination of SV2A.

### 3.6. Real Sample Analysis

To evaluate the reliability of the constructed sensor in routine analysis, the standard addition method was employed to detect SV2A in venous serum from healthy C57 mice using the prepared sensor. Concurrently, commercial SV2A ELISA kits were used to analyze the actual samples. The practical detection capability of the immunosensor for serum samples was evaluated, and the results are presented in Table 3. The recoveries of the sensor ranged from 98% to 109%, which falls within the acceptable range. Correlation analysis revealed a high degree of correlation between the two methods (R^2^ = 0.998), and a paired t-test indicated no significant difference (*p* > 0.05), demonstrating the excellent accuracy of the proposed immunosensor.

## 4. Conclusions

This study presents an effective electrochemical strategy for the rapid detection of the Alzheimer’s disease (AD) biomarker, synaptic vesicle glycoprotein 2A (SV2A). A 3-COOH-PPy/GO nanocomposite was successfully synthesized via the electropolymerization of 3-COOH-PPy onto graphene oxide (GO). Capitalizing on the synergistic interplay between the large specific surface area of GO and the superior conductivity of 3-COOH-PPy, the composite established an optimal interface for biomolecule immobilization and electron transfer, thereby significantly amplifying the electrochemical response. Furthermore, an HRP-catalyzed TMB/H_2_O_2_ system was employed as an enzyme-mediated signal amplification strategy to ensure high sensitivity. The developed immunosensor demonstrated excellent analytical performance, characterized by a wide linear range (2 ng/mL to 16 μg/mL), high sensitivity, robust selectivity, and stability, with a limit of detection (LOD) of 0.15 ng/mL. The practical applicability of the method was validated by detecting SV2A in mouse serum samples, yielding recovery rates between 98% and 109%. By integrating the merits of nanomaterial modification and enzyme-mediated amplification, this immunosensor offers a sensitive and reliable platform for the early diagnosis of AD. Future research will focus on integrating microfluidic technology to streamline operational procedures, aiming to translate this platform into a practical point-of-care testing (POCT) device for clinical applications.

## Figures and Tables

**Table 1:** Comparison of A_eff_ and Rct for Different Electrodes.

Electrode	A_eff_/(cm^2^)	Rct/(Ω)
GO/GCE	0.1141	774.0
3-COOH-PPy/GCE	0.3369	44.8
3-COOH-PPy/GO/GCE	0.3872	35.3
Ab1/3-COOH-PPy/GO/GCE	0.2469	528.4
BSA/Ab1/3-COOH-PPy/GO/GCE	0.2075	937.0
SV2A/BSA/Ab1/3-COOH-PPy/GO/GCE	0.1780	1330.0
Ab2/SV2A/BSA/Ab1/3-COOH-PPy/GO/GCE	0.1615	2800.0

Note: Electrochemical effective active area (A_eff_). Charge-transfer resistance (Rct).

**Table 2:** Comparison of Performance of Different Methods for Constructing Immunosensors Using Graphene.

Methods	Electrode	Linear Range	LOD	Recognition Element	Real Sample	Reference
Voltammetric sensor	PPy-GO/GCE	2–14 ng/mL	1.63 ng/mL	Anti-HCV	Serum	[31]
Voltammetric sensor	PtNPs/GO-COOH	3–30 ng/mL	1.22 ng/mL	AFP	Serum	[32]
Voltammetric sensor	GO/PAA/SPCE	1–100 ng/mL	0.54 ng/mL	IgG	Serum	[33]
Amperometric sensor	ITO-PET/ZnO NRs/rGO	10–1000 ng/mL	1.25 ng/mL	H-IgG	Serum	[34]
Voltammetric sensor	3-COOH-PPy-GO/GCE	2 ng/mL–16 μg/mL	0.15 ng/mL	SV2A	Serum	This work

**Table 3:** Measurement results of SV2A protein recovery rate by 3-COOH-PPy/GO/GCE.

Sample	Added (ng/mL)	Measured (ng/mL)	Recovery%	RSD%	ELISA		RSD%	t
					Measured (ng/mL)	Recovery%		
Mouse serum	4	3.92	98.09	5.00	4.21	105.23	4.79	1.61
		4.05	101.22		4.58	114.42		
		4.32	108.11		4.23	105.64		
	8	8.16	102.04	4.25	8.42	105.29	1.65	1.42
		7.91	98.82		8.44	105.50		
		8.60	107.47		8.67	108.42		
	12	12.17	101.46	3.16	12.35	102.94	2.52	1.02
		12.82	106.86		12.00	100.01		
		12.90	107.51		12.62	105.17		

## Data Availability

The original contributions presented in this study are included in the article. Further inquiries can be directed to the corresponding authors.
